# Publisher Correction: Low genetic variation is associated with low mutation rate in the giant duckweed

**DOI:** 10.1038/s41467-019-09796-5

**Published:** 2019-04-16

**Authors:** Shuqing Xu, Jessica Stapley, Saskia Gablenz, Justin Boyer, Klaus J. Appenroth, K. Sowjanya Sree, Jonathan Gershenzon, Alex Widmer, Meret Huber

**Affiliations:** 1 Institute for Evolution and Biodiversity, University of Münster, Hüfferstrasse 1, 48149 Münster, Germany; 2 Center for Adaptation to a Changing Environment, ETH Zurich, Universitätstrasse 16, 8092 Zürich, Switzerland; 3 Department of Biochemistry, Max Planck Institute for Chemical Ecology, Hans-Knöll-Strasse 8, 07745 Jena, Germany; 4 Matthias-Schleiden-Institute, Plant Physiology, Friedrich Schiller University of Jena, Dornburgerstraße 159, 07743 Jena, Germany; 5 Department of Environmental Science, Central University of Kerala, Periye, 671316 India; 6 Institute of Integrative Biology, ETH Zurich, Universitätstrasse 16, 8092 Zürich, Switzerland; 7 Institute of Plant Biology and Biotechnology, University of Münster, Schlossplatz 7, 48143 Münster, Germany

Correction to: *Nature Communications* 10.1038/s41467-019-09235-5, published online 18 March 2019

The original HTML version of this Article had an incorrect Published online date of 20 March 2019; it should have been 18 March 2019. This has been corrected in the HTML version of the Article. The PDF version was correct from the time of publication.

