# Supplementary material for: Low genetic variation is associated with low mutation rate in the giant duckweed
Source: Nat Commun. 2019 Mar 18;10:1243. doi: 10.1038/s41467-019-09235-5 (PMC6423293; doi:10.1038/s41467-019-09235-5)
Supplement: Supplementary file 1 — Supplementary Information [file 41467_2019_9235_MOESM1_ESM.pdf]

Supplementary Information

Low genetic variation is associated with low mutation rate in the giant duckweed

Xu et al.

Supplementary Figures

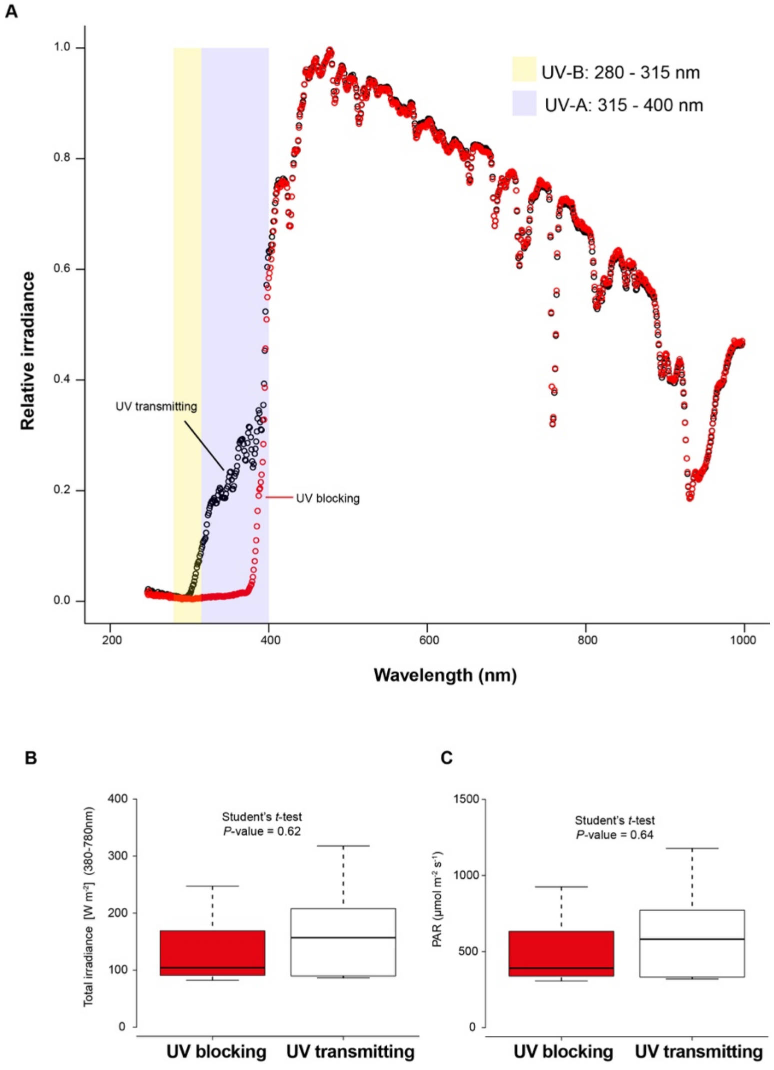

Supplementary Figure 1. Spectral properties of UV blocking and UV transmitting plexiglass covers. A, relative irradiance (irradiance / max irradiance per plate) in UV transmitting (GS 2458, *n* = 9) and UV blocking (UV Gallery100, *n* = 9) plexiglass. B and C, total irradiance (B) and PAR (C) did not differ between UV transmitting and UV blocking plexiglass (*n* = 9). The irradiance from 250 to 1000 nm, as well as total irradiance between 380 and 780 nm and photosynthetic active radiation (PAR) between 400 and 700 nm were measured to assess the spectrum of the UV transmitting and UV blocking types of plexiglass (Sandrock, Germany) that were used in the mutation accumulation experiments.

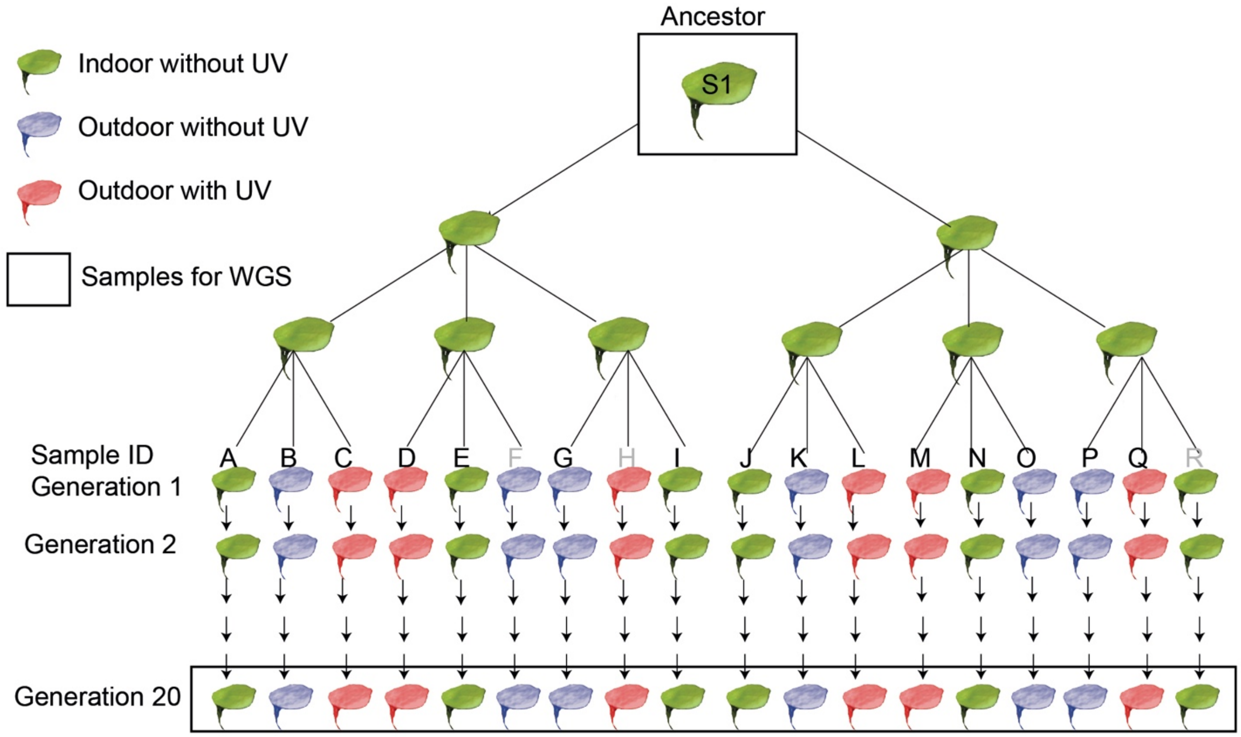

Supplementary Figure 2. Propagating scheme of the individuals for the mutation accumulation experiments. A single ancestor was used to propagate 18 individuals, which were then propagated for 20 generations with a single-descendant approach. Among the 18 individuals collected, three individuals (F, H and R, in gray color) were not included for the data analysis due to their low sequencing depth. Each color represents a different treatment. Samples that were used for sequencing are marked with a black box (bottom row).

Supplementary Tables

Supplementary Table 1. Summary statistics of population genomics in *S. polyrhiza*. π refers to the estimated pairwise nucleotide diversity. π_a refers to the nucleotide diversity at non-synonymous sites and π_s refers to the nucleotide diversity at synonymous sites.

**Summary statistics**	**Nonsynonymous variants**	**Synonymous variants**	**Genome-wide *π***	***π* in coding regions**	***π* in intergenic regions**	**πN**	**πS**	**πN/πS**	**π_intron**
All populations combined	14,191	8,865	0.0015	0.0006	0.0016	0.0004	0.0009	0.44	0.0008

Supplementary Table 2. Pairwise Fst between four population groups. The number of sequenced individuals is listed in bracket.

**Population**	India (13)	SE Asia (18)	Europe (27)	America (10)
India (13)	/	/	/	/
SE Asia (18)	0.47	/	/	/
Europe (27)	0.65	0.35	/	/
America (10)	0.82	0.67	0.79	/

Supplementary Table 3. Summary of nucleotide diversity in *S. polyrhiza*. The summary statistics of nucleotide diversity are shown separately for each chromosome. Ti/Tv: Transition to Transversion ratio. Fst is calculated among all four populations. π: average pairwise nucleotide diversity from all sites.

Chr ID	Chromosome size (bp)	Callable sites (bp)	Ti/Tv	Fst	π				Tajima's D			
					India	SE Asia	Europe	America	India	SE Asia	Europe	America
ChrS01	11466534	10230868	2.16	0.67	0.00030	0.00057	0.00034	0.00020	0.34	0.22	0.11	0.34
ChrS02	8941172	8118899	2.21	0.67	0.00030	0.00064	0.00038	0.00017	-0.16	0.07	0.14	0.63
ChrS03	8796147	8025920	2.23	0.73	0.00030	0.00069	0.00039	0.00016	-0.16	0.36	-0.22	0.35
ChrS04	8491500	7618096	2.18	0.69	0.00028	0.00058	0.00040	0.00013	-0.13	-0.35	0.56	0.15
ChrS05	8389602	7362890	2.28	0.66	0.00048	0.00070	0.00044	0.00024	0.15	0.08	-0.41	0.55
ChrS06	8130874	7068555	2.24	0.71	0.00026	0.00066	0.00038	0.00020	-0.26	0.33	0.03	0.89
ChrS07	8107549	7057007	2.30	0.70	0.00047	0.00068	0.00027	0.00019	0.23	0.28	-0.55	0.80
ChrS08	7340019	6432486	2.09	0.68	0.00032	0.00056	0.00031	0.00016	-0.09	0.00	0.32	0.40
ChrS09	7208038	6432306	2.19	0.68	0.00038	0.00063	0.00040	0.00019	0.08	0.05	0.57	0.90
ChrS10	7041313	6114959	2.25	0.66	0.00041	0.00065	0.00048	0.00025	0.36	0.07	0.51	0.61
ChrS11	6552830	5535985	2.20	0.71	0.00030	0.00061	0.00033	0.00019	-0.02	0.10	-0.03	0.84
ChrS12	5946178	5212165	2.18	0.62	0.00042	0.00076	0.00049	0.00027	0.13	0.25	-0.02	0.96
ChrS13	5476630	4837586	2.20	0.70	0.00027	0.00053	0.00041	0.00018	-0.19	0.25	0.02	0.92
ChrS14	5103705	4493067	2.19	0.63	0.00039	0.00069	0.00044	0.00028	0.27	-0.21	0.28	-0.08
ChrS15	4726429	3824613	2.38	0.65	0.00039	0.00069	0.00043	0.00022	-0.22	0.42	0.20	0.77
ChrS16	4623610	4102426	2.40	0.72	0.00031	0.00080	0.00033	0.00023	-0.53	0.13	-0.73	1.1
ChrS17	4564609	3967959	2.30	0.67	0.00043	0.00078	0.00042	0.00018	0.35	0.40	0.12	0.31
ChrS18	4370269	3888065	2.31	0.71	0.00034	0.00064	0.00045	0.00015	-0.53	-0.24	0.09	0.49
ChrS19	3727809	3123686	2.26	0.65	0.00043	0.00066	0.00044	0.00018	0.48	0.12	0.88	0.90
ChrS20	3541257	3201853	2.11	0.69	0.00034	0.00070	0.00033	0.00023	0.43	0.20	-0.29	0.12

Supplementary Table 4. Summary of the information of the sequencing coverage for the mutation accumulation experiments. The coverage was calculated based on all properly mapped reads after removing the PCR duplicates. All sample IDs refer to the samples showed in Supplementary Figure 2, except sample V, which refers to the ancestor (labeled as S1 in Supplementary Figure 2).

Sample ID	Coverage
A	29
B	27
C	30
D	36
E	22
G	33
I	34
J	32
K	20
L	24
M	34
N	28
O	23
P	27
Q	23
V	29
