# Supplementary material for: Low genetic variation is associated with low mutation rate in the giant duckweed
Source: Nat Commun. 2019 Mar 18;10:1243. doi: 10.1038/s41467-019-09235-5 (PMC6423293; doi:10.1038/s41467-019-09235-5)
Supplement: Supplementary file 3 — Description of Additional Supplementary Files [file 41467_2019_9235_MOESM3_ESM.pdf]

Description of Additional Supplementary Files

Supplementary Data 1. Sample and sequencing information of 68 *S. polyrhiza* genotypes. Mapped reads refer to all uniquely mapped reads, coverage was calculated based on the nuclear genome, and accession ID refers to the registered four-digit code of each genotype. The relatively low mapping rates from some samples were mainly due to non-plant DNAs.

Supplementary Data 2. Annotation of SNPs at the gene level. The total number of SNPs that were found in each gene is listed according to the predicted effects.

Supplementary Data 3. Summary of mutation rate and effective population size (N_e) estimates. Data is obtained from Lynch et al.[1], with a few updates [2]. Mutation rate (μ) is listed as per generation per site. NA: not available. CDS: protein coding sequence. The estimated π from neutral sites is calculated as D × N_e μ × where D refers to 4 (diploid species) or 2 (haploid species).

1. Lynch M, et al. Genetic drift, selection and the evolution of the mutation rate. *Nat Rev Genet* **17**, 704-714 (2016).

2. Feng CG, et al. Moderate nucleotide diversity in the Atlantic herring is associated with a low mutation rate. *eLife* **6**, e23907 (2017).

Supplementary Data 4. Climate and light spectrum information in Jena (Germany), at the place where the outdoor mutation accumulation experiments were performed. Ambient temperature, global radiation, PAR radiation and the UV spectrum are shown hourly.

Supplementary Data 5. Detailed information for all putative MA variants. Most of the putative variants were loss-of-heterozygosity (LOH) mutations and located in clusters, likely due to false positives. The variants that were validated using Sanger sequencing are highlighted in red, which are all located in non-coding region. AD refers to the number of reads supporting reference and alternative alleles, respectively.

Supplementary Data 6. Primer information for validating the variants. All primers that were used for validating the candidate variants are shown. Primer sequence information is shown in forward (F) and reverse (R). The validation results are indicated in bold text.
