# Supplementary material for: Low genetic variation is associated with low mutation rate in the giant duckweed
Source: Nat Commun. 2019 Mar 18;10:1243. doi: 10.1038/s41467-019-09235-5 (PMC6423293; doi:10.1038/s41467-019-09235-5)
Supplement: Supplementary file 10 — Reporting Summary [file 41467_2019_9235_MOESM10_ESM.pdf]

nature research

Corresponding author(s): Shuqing Xu & Meret Huber

Reporting Summary

Nature Research wishes to improve the reproducibility of the work that we publish. This form provides structure for consistency and transparency in reporting. For further information on Nature Research policies, see Authors & Referees and the Editorial Policy Checklist.

Statistical parameters

When statistical analyses are reported, confirm that the following items are present in the relevant location (e.g. figure legend, table legend, main text, or Methods section).

n/a	Confirmed	
☐	☒	The exact sample size (*n*) for each experimental group/condition, given as a discrete number and unit of measurement
☐	☒	An indication of whether measurements were taken from distinct samples or whether the same sample was measured repeatedly
☒	☐	The statistical test(s) used AND whether they are one- or two-sided<br>*Only common tests should be described solely by name; describe more complex techniques in the Methods section.*
☒	☐	A description of all covariates tested
☐	☒	A description of any assumptions or corrections, such as tests of normality and adjustment for multiple comparisons
☒	☐	A full description of the statistics including central tendency (e.g. means) or other basic estimates (e.g. regression coefficient) AND variation (e.g. standard deviation) or associated estimates of uncertainty (e.g. confidence intervals)
☒	☐	For null hypothesis testing, the test statistic (e.g. *F*, *t*, *r*) with confidence intervals, effect sizes, degrees of freedom and *P* value noted<br>*Give P values as exact values whenever suitable.*
☒	☐	For Bayesian analysis, information on the choice of priors and Markov chain Monte Carlo settings
☒	☐	For hierarchical and complex designs, identification of the appropriate level for tests and full reporting of outcomes
☒	☐	Estimates of effect sizes (e.g. Cohen's *d*, Pearson's *r*), indicating how they were calculated
☐	☒	Clearly defined error bars<br>*State explicitly what error bars represent (e.g. SD, SE, CI)*

Our web collection on statistics for biologists may be useful.

Software and code

Policy information about availability of computer code

Data collection	Sequencing data were collected from Illumina
Data analysis	The softwares that were used for data analysis were described in the Method section. Scripts that were used for variants filtering are available upon request.

For manuscripts utilizing custom algorithms or software that are central to the research but not yet described in published literature, software must be made available to editors/reviewers upon request. We strongly encourage code deposition in a community repository (e.g. GitHub). See the Nature Research guidelines for submitting code & software for further information.

Data

Policy information about availability of data

All manuscripts must include a data availability statement. This statement should provide the following information, where applicable:

- Accession codes, unique identifiers, or web links for publicly available datasets
- A list of figures that have associated raw data
- A description of any restrictions on data availability

All raw DNA sequences obtained in this study are submitted to NCBI under Bioproject PRJNA476302. Source data for figure(s) are provided with the paper. A reporting summary for this Article is available as a Supplementary Information file.

Field-specific reporting

Please select the best fit for your research. If you are not sure, read the appropriate sections before making your selection.

☐ Life sciences ☐ Behavioural & social sciences ☒ Ecological, evolutionary & environmental sciences

For a reference copy of the document with all sections, see nature.com/authors/policies/ReportingSummary-flat.pdf

Ecological, evolutionary & environmental sciences study design

All studies must disclose on these points even when the disclosure is negative.

Study description	We investigated genetic diversity and spontaneous mutation rated in the giant duckweed (Spirodela polyrhiza).
Research sample	Research samples are Spirodela polyrhiza.
Sampling strategy	This has been described in details in the method section.
Data collection	Whole genome sequencing data of 68 genotypes was obtained from Illumina X10 in BGI (China), and the sequencing data for the MA experiments are from Illumina HiSeq 4000 at the Genomics Center of the Max Planck Institute for Plant Breeding Research in Cologne (Germany).
Timing and spatial scale	The genomes of 68 genotypes were sequenced on 4th January 2017. The genomes of S. polyrhiza in the MA experiments were sequenced in two batches. The first batch was sequenced on the 23rd February 2017 and the second batch was sequenced on the 3rd May 2017.
Data exclusions	We initially sequenced 71 individuals that were identified as S. polyrhiza based on morphology. However, genomic information showed that three samples were other species (extremly low mapping rate to S. polyrhiza refernce genome). Therefore, we excluded these three individuals in our data analysis. For the MA experiments, we initially sequenced 18 samples. Three samples had very low coverage and therefore were removed from our analysis.
Reproducibility	Our manuscript has two major results: low genetic diversity and low mutation rate in S. polyrhiza. For the first result, we sequenced additional 40 genotypes (as a follow-up project) that were collected in Europe and Asia. The results showed a very similar pattern, which suggests that the low genetic diversity in S. polyrhiza is reproducible. The results will be reported in our following manuscript. For the low mutation rate, repeating the whole experiments is very time consuming and expensive. However, the identified mutation rates were similar among different treatments, indicating the results are robust. In addition, we explored different variant filtering parameters in our data analysis and all showed very similar results.
Randomization	For the MA experiments, samples and treatments were randomized.
Blinding	Blinding was not relevant in our study.

Did the study involve field work? ☒ Yes ☐ No

Field work, collection and transport

Field conditions	Detailed information is provided in supplementary dataset 2.
Location	For the outdoor MA lines, plants were moved at the end of June 2016 into a sun-exposed field site in Jena, Germany (50° 53’06.7’’N 11°40’53.1’’E).
Access and import/export	Not relevant in our study.
Disturbance	Not relevant in our study.

Reporting for specific materials, systems and methods

Materials & experimental systems

n/a	Involved in the study
☒	☐ Unique biological materials
☒	☐ Antibodies
☒	☐ Eukaryotic cell lines
☒	☐ Palaeontology
☒	☐ Animals and other organisms
☒	☐ Human research participants

Methods

n/a	Involved in the study
☒	☐ ChIP-seq
☒	☐ Flow cytometry
☒	☐ MRI-based neuroimaging
